# Innate Immune System and Preeclampsia

**DOI:** 10.3389/fimmu.2014.00244

**Published:** 2014-05-26

**Authors:** Alejandra Perez-Sepulveda, Maria Jose Torres, Maroun Khoury, Sebastian E. Illanes

**Affiliations:** ^1^Centro de Investigaciones Biomédicas, Faculty of Medicine, Universidad de los Andes, Santiago, Chile; ^2^Cells for Cells, Santiago, Chile

**Keywords:** preeclampsia, mesenchymal stem cells, immunomodulation, Th1–Th17, Th2-Treg

## Abstract

Normal pregnancy is considered as a Th2 type immunological state that favors an immune-tolerance environment in order to prevent fetal rejection. Preeclampsia (PE) has been classically described as a Th1/Th2 imbalance; however, the Th1/Th2 paradigm has proven insufficient to fully explain the functional and molecular changes observed during normal/pathological pregnancies. Recent studies have expanded the Th1/Th2 into a Th1/Th2/Th17 and regulatory T-cells paradigm and where dendritic cells could have a crucial role. Recently, some evidence has emerged supporting the idea that mesenchymal stem cells might be part of the feto-maternal tolerance environment. This review will discuss the involvement of the innate immune system in the establishment of a physiological environment that favors pregnancy and possible alterations related to the development of PE.

## Introduction

Preeclampsia (PE), its complications and associated pathologies, have become one of the main causes of maternal and fetal morbidity and mortality in the world (1), causing nearly 40% of premature births delivered before 35 weeks of gestation and complicating around 2–8% of all pregnancies worldwide. Moreover, PE has been strongly associated with an increased risk of later-life death due to cardiovascular disease, independent of other risk factors (2–4).

Preeclampsia is classically defined as the new onset of hypertension during the second half of pregnancy accompanied by significant proteinuria (5). Despite the breakthroughs in the understanding of PE’s etiopathogenesis, the physiopathology that triggers the disease is still not clearly elucidated. Nevertheless, it seems clear that the development of PE requires the presence of a placenta, since the clinical syndrome will not develop in the absence of a placenta and it disappears soon after placental delivery (6). It is also widely accepted that the pathophysiological process of PE begins with an abnormal trophoblast invasion early in pregnancy, which produces increased placental oxidative stress contributing to the development of systemic endothelial dysfunction in the later phases of the disease. This leads in turn to the characteristic clinical manifestations of PE.

## Etiopathogenesis of PE: A Silencing Start Early in Pregnancy

During the first weeks of a normal gestation, after the blastocyst makes contact with the maternal decidua, cytotrophoblast cells proliferate forming cell columns intruding maternal tissue (7). From the tip of these anchoring villous structures, extravillous trophoblast (EVT) cells derived from this proliferating cytotrophoblast, invade the maternal decidua differentiating further into interstitial and endovascular trophoblast cells. The invasion process begins at the center of the placental bed, and expands progressively to the lateral areas, like a ring-shape spread. During the interstitial invasion, the compact decidual tissue is “swamped” by interstitial EVT cells that, from 8 weeks onward, can be seen both in the inner myometrium zone of the placenta – where they stop the invasive process – and clustered around blood vessels (8). At the same time, endovascular trophoblast cells migrate into the maternal spiral arteries in order to plug these vessels. Around 10–12 weeks of gestation, trophoblast plugs begin to dissolve and endovascular trophoblast replace maternal endothelial lining as far as the inner third of myometrium, degrading the muscular and elastic component of the vessel walls resulting in the formation of low-resistance vessels that are required for adequate uteroplacental circulation and fetal growth (7, 9). Thus, a new onset of maternal blood flow into the intervillous space begins. A deficient trophoblast invasion process and failures in the spiral artery remodeling transformation have been demonstrated to be associated with the development of placental diseases such as PE (10, 11), but the trigger of these altered processes is still not well understood.

Regarding abnormal trophoblast invasion process, in PE the maternal vessels, such as spiral arteries, are poorly remodeled. In these altered vessels, the diameter is diminished in comparison with normal remodeled vessels, and also the extent of remodeling process is decreased. Further, the vascular smooth muscle layer remains surrounding PE remodeled vessels, contributing to a contractile tone of these arteries. This observation is in accordance to the idea that a maternal pulsatile blood flow to the placental bed could induce hypoxia–reperfusion events that can be related to placental hypoxia, and placental oxidative stress observed in PE (12).

The trophoblast invasion process and finally the successful in pregnancy establishment relies on an orchestrated interaction between trophoblast-derived cells and maternal tissue that is crucial for normal pregnancy and that might give clues for the understanding of PE development. In this regard, the maternal immune system plays a key role, allowing the interaction of two immunologically different beings, the embryo and mother.

## Preeclampsia Development and the Immune System

Several hypotheses have been proposed to explain the abnormal trophoblastic invasion early in pregnancy associated with PE, many of them suggesting that it might be triggered by an altered maternal immune response or a defective development of maternal tolerance to the semi-allogeneic fetus (13–17). Epidemiological evidence supporting this idea has been published by many groups (18–20), suggesting the importance of the maternal immune system in the pathogenesis of PE.

In order to elucidate if the deficient invasion of trophoblast observed in PE might be due to an alteration of the immune-tolerance environment in the decidua, different studies have been performed in order to characterize the immune milieu of these patients. An excessive activation of neutrophils and monocytes in PE patients (circulating and in the decidua) have been described by many groups (21–26). These monocytes have been found to spontaneously synthesize greater amounts of pro-inflammatory cytokines such as IL-1b, IL-6, and IL-8 (27). Furthermore, CD4^+^ and CD8^+^ T-lymphocytes along with natural killer (NK) cells and dendritic cells (DCs) have also been found to respond differently in PE women compared to normal pregnancies, tending to a pro-inflammatory response, similar to that seen in non-pregnant women, instead of the immunotolerant and anti-inflammatory response seen in normal pregnancies (28–30). Moreover, DCs demonstrate a pro-inflammatory bias secondary to dysregulation of toll-like receptors (TLRs) (31) and decidual NK cells, which play a particularly important role in regulating cellular interactions in successful placentation by promoting placental development and maternal decidual spiral artery modifications, are found to secrete lower amounts of invasion-promoting factors when taken from decidual tissue from women with altered uterine artery Doppler (non-invasive screening for PE development) (17).

## Preeclampsia: A Th1–Th17/Th2-Treg Imbalance

**Figure 1:**
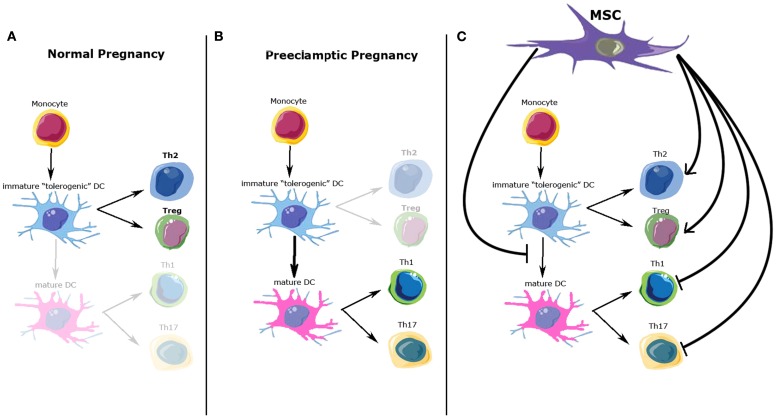
**Possible immunomodulatory role of mesenchymal stem cells (MSC) over immune cells involved in normal and preeclamptic pregnancy**. **(A)** Normal pregnancy is considered as a Th2 type immunological state, where Th2 CD4^+^ T-cells and Treg cells response and cytokine profile predominate. **(B)** On the other hand, preeclamptic pregnancies have been considered as a maternal pro-inflammatory state with Th1/Th17 predominance. **(C)** Possible MSC effects over the immune cell types involved in normal and preeclamptic pregnancies. MSC inhibit maturation of dendritic cells, maintaining a tolerogenic DC phenotype; MSC inhibit Th1/Th17 proliferation and function, whiles promote Treg and Th2 differentiation and cytokine secretion. All these effects favor a Th2/Treg phenotype. DCs, dendritic cells; Th2, T-helper 2; Th1, T-helper 1; Th17, T-helper 17; Treg, T-regulators cells.

Another important immune aspect of PE development is the Th1/Th2 imbalance. Normal pregnancy is considered to be a Th2 type immunological state, which favors an immunotolerant environment for the prevention of fetal rejection (32) (Figure 1A). On the other hand, PE pregnancies have been characterized as a maternal pro-inflammatory state with Th1 predominance: increased plasma levels of pro-inflammatory cytokines have been described by different authors, mainly during the second and third trimester of pregnancy (33, 34) (Figure 1B). However, the Th1/Th2 paradigm has been proven incomplete to fully explain the functional and molecular changes observed during normal/pathological pregnancies. Recent studies have described several other immune cells involved in this process, expanding the Th1/Th2 paradigm into the Th1/Th2/Th17 and regulatory T cells (Treg) paradigm, introducing Treg as regulators of Th17 lymphocytes and other immune cell types involved in the feto-maternal tolerance (28, 35).

Th17 cells, a relatively novel CD4^+^ lymphocyte subpopulation associated with Th1 cytokine profile, are characterized by the production of IL-17. An up-regulation of this lymphocyte subpopulation has been related with the development and progression of autoimmune and chronic inflammatory diseases, allergic disorders, and graft-rejection reactions (36). Furthermore, Th17 subpopulation has been described as up-regulated in PE compared to normal pregnancy. Darmochwal-Kolarz et al. have reported that, IL-17-producing lymphocytes are increased in peripheral blood of PE patients in the third trimester of pregnancy, compared to a control group. Moreover, they described a significant correlation between Th17, IL-2- and IFN-g-producing T-cells, and PE development (37). Their data firmly support the idea that the up-regulation of Th17 immunity is related to the activation of a Th1 response in PE, suggesting that regulatory role of Treg could be also altered.

Regulatory T cell is another lymphocyte subpopulation, characterized by the expression of a high level of CD25, cytotoxic T-lymphocyte antigen 4 (CTLA-4), and the expression of the transcription factor FOXP3 (38). Treg plays a crucial role in the development and maintenance of tolerance in peripheral tissues, as well as in the induction of transplantation tolerance, so it has been also proposed as a key factor in the maintenance of materno-fetal tolerance (39–41). A low amount and activity of Treg cells has been described in PE, while normal human pregnancy is associated with elevated numbers and immune suppressive effects of these cells (42). Peripheral Treg cells are normally produced within peripheral tissues, such as decidualized endometrium during early pregnancy, and respond to antigens specifically restricted to the tissue where they are found (43–45). Peripheral Treg must meet antigens presented by “tolerogenic” DCs in an appropriate cytokine environment to proliferate, get to functional maturity, and exert their suppressive effects. Tolerogenic DCs are characterized by their immature or semi-mature phenotype, their altered expression of co-stimulatory molecules CD80 and CD86, and the lack of expression of the Th1-inducing cytokine IL-12 (46). Only these DCs possess the functional characteristics of immature DCs and consistently induce Treg cells with immunosuppressive function (Figures 1A,B).

An important cell type that induces and maintains the tolerogenic phenotype of DCs, are mesenchymal stem cells (MSC) (47). Furthermore, a growing body of evidence supports the idea that MSC can modulate the behavior and cytokine secretion of all cell types previously described involved in feto-maternal tolerance development (48), suggesting a plausible role for MSC in the regulation of trophoblast invasion, and conversely a potential role in abnormal placentation, a feature of PE.

## Mesenchymal Stem Cells: A Cell with Important Immunomodulatory Potential

Mesenchymal stem cells are multipotent mesenchymal stromal cells that proliferate *in vitro* as plastic-adherent cells, have fibroblast-like morphology and can differentiate into bone, cartilage, and fat cells (49). They are found in almost all human tissues and an endometrial mesenchymal stem cells (eMSC) population has also been identified by Gargett et al. These eMSC show high clonogenic properties similar to bone marrow-derived MSC (BM-MSC) (50, 51). Recent studies have described the influencing MSC capacities over immune and inflammatory responses, and especially in the endometrium these cells could be key players in the immune regulation needed for a successful implantation and normal invasion process carried out by the trophoblast. Inversely, an abnormal performance of these cells at this crucial point could lead to an abnormal development of the trophoblast and an impaired placentation.

It has been shown that MSC suppress the differentiation of DCs from monocytes by arresting them in G_0_ phase of cell cycle, an effect that is mediated by soluble factors (47). Moreover, MSC interfere in maturation of DCs avoiding a Th1 response typical of mature DCs, and promoting an immature DC phenotype that helps to generate a tolerogenic environment (52). Besides, Jiang et al. have reported that MSC maintain DC in an immature state and that MSC inhibit up-regulation of IL-12p70, a pro-inflammatory cytokine (53). Similarly, it has been reported that MSC can alter the cytokine profile secreted by DC to induce a tolerogeneic microenvironment (54). Specifically, MSC induce the DC-associated production of IL-10, which in turn, induce the secretion of the anti-inflammatory cytokine IL-4 by Th2 cells (55). All these effects depend on the cytokine environment, because it has been shown that an increase of pro-inflammatory cytokines, such as IL-6 and TNF-a, reverse the immunosuppressive effects of MSC over DC cells (47).

Also, it has been described that MSC inhibit Th17 differentiation and function, decreasing the number and activity of these cells in the inflammation site. Moreover, it has been shown that the co-incubation of MSC with Th17 induces “regulatory” features in these cells even in an inflammatory environment. This effect is carried out by the down-regulation of retinoic-acid-receptor-related orphan receptor gamma t (RORgt) transcription factor and by the up-regulation of FOXP3 transcription factor (56). These effects could be associated to the release of soluble factors from MSC, such as prostaglandin E2 (PGE_2_), or by the modification of cytokine environment that favors a Treg phenotype (57).

Furthermore, it has been shown that MSC increase the number and the activity of Treg (58). It has been demonstrated that co-culture of CD4^+^ T-cells with MSC induce the appearance of FOXP3^+^CD25^High^ T-cells. Another possible mechanism for the increase of Treg number by MSC is the inhibition of IL-6 production, which is a necessary cytokine in the Th17 differentiation process from Tregs. Also, it has been shown that the influence of MSC over DC cells favors the generation of Treg cells, because of the tolerogenic environment generated both by MSC and by tolerogenic DCs (47, 54).

Mesenchymal stem cell not only can influence the phenotype of the different cells that play a role in the immune environment of pregnancy, but also have a role in the regulation of Th1/Th2 balance. It has been shown that MSC can shift a Th1 phenotype to a Th2. This effect could be performed through the modulation of DC phenotype (shifting from a DC1 or Th1-associated phenotype to a DC2 or Th2-associated phenotype) or by the direct effects over Th1/Th2 cells. In this regard, MSC inhibit CD4^+^ T-cell proliferation by the inhibition of the entry to S phase of cell cycle (47). This effect is mediated at least in part by soluble factors such as TGF-β, hepathocyte growth factor (HGF), and PGE_2_ (54, 59). MSC inhibit proliferation of activated T-cells in respond to: (i) non-specific stimuli such as DCs, phytohemagglutinin (PHA), and IL-2, (ii) their specific antigen (55). MSC also inhibit Th1 phenotype by the inhibition of IFN-g production, which is necessary for Th1 cells development, and by increasing Treg cell number, that works as a counterpart of Th1 cells. MSC not only suppress Th1 response, but favors the emergence and maintenance of Th2 response by inducing IL-4 production that favor the Th2 differentiation (54, 55, 57). There are several studies that indicate that MSC could positively alter the Th1/Th2 balance. Bai et al., showed in an experimental allergic encephalomyelitis model that MSC induce neurological improvements by the reduction of T-cells infiltration to the brain and by the increased production of Th2 cytokines such as IL-4 and IL-5 production accompanied by the reduction in Th1/Th17 related cytokines such as IL-17 IFN-γ and TNF-α (60).

In summary, MSC regulate immune cell types involved in the feto-maternal tolerance that allows a normal invasion of the decidua by the EVT (Figure 1C). Dysregulation of this invasive process is part of the etiopathogenesis of PE, but clear evidence of the involvement of the immunomodulatory properties of eMSC in this process remains to be elucidated.

## Molecular Mechanisms of MSC Immunosuppressive Effect

So far, we have discussed about MSC effects on different immune cell types and its potential role in the abnormal placentation observed in patients that develop PE, but the mechanisms underlying these effects need to be explained. Inhibitory effects of MSC over T-cell proliferation could be accomplished by at least two different ways.

### Cell-to-cell contact-dependent mechanism

It is mediated mainly by PD1–PD1L pathway (61). PD-L1 is a transmembrane glycoprotein and a ligand of the programed cell death protein 1 (PD-1) that is expressed in various cell types, including T-cells, macrophages, DCs, and placenta (62, 63). The interaction of PD-L1 with PD-1 leads to the suppression of the immune response (62). PD-L1 is considered a key suppressor factor in maternal tolerance (64). It has been shown that PD-L1 is up-regulated on decidual T-cells during pregnancy (65), and that their expression on the surface of Tregs is essential to exert their suppressive effect and to control the maternal immune response (66). Moreover, placental MSC express higher levels of PD-L1 than BM-MSC, although IFN-γ treatment proved to have a lower immunomodulatory capacity on T-cell proliferation (67). Furthermore, PD-L1 pathway in BM-MSC mediates suppression of Th17 cell proliferation and IL-17 production (68). However, there is no data to the best of our knowledge about the expression of PD-L1 on the decidua and eMSC of patients that develop PE.

### Metabolism of the essential amino acid tryptophan

Mesenchymal stem cells express the enzyme indoleamine 2,3-dioxygenase (IDO), a tryptophan-degrading enzyme, that through the consumption of tryptophan amino acid serves as a natural immunoregulatory mechanism for the inhibition of T-cell proliferation. Munn et al. showed that the functional inhibition of IDO resulted in the uniform rejection of allogeneic fetuses, suggesting the crucial role of this enzyme in maternal tolerance maintenance (69). Similarly, it has been shown that placental MSC treated with IFN-g showed an increase in IDO expression, inhibiting autologous T-cell proliferation (70). In PE, IDO expression is increased (71) and this altered IDO expression has been postulated to be associated with the reduction of Treg cell subset, a feature observed in patients that develop PE (72).

On the other hand, MSC can produce immunosuppressive effects by the production and release of immunosuppressive factor such as HLA-G and PGE_2_.

- *HLA-G*: it has been shown that MSC express and secrete HLA-G (73, 74). This expression can be up-regulated by progesterone treatment (75) and pro-inflammatory cytokines (76). Furthermore, the induction of HLA-G expression as a strategy to enhance the immunosuppressive properties of MSC in transplantation has been postulated (77). HLA-G is a non-classical MHC class Ib molecule that initially was identified in trophoblast cells. HLA-G has soluble and membrane-bound isoforms (78, 79), and it is recognized by immunoglobulin-like transcript receptor expressed in T-cells, B cells, NK cells, and macrophages (79). The physiological role of HLA-G during pregnancy is to establish immune-tolerance at the maternal-fetal interface, abrogating the cytolytic activity of maternal NK and cytotoxic T-cells against fetal tissue (80). HLA-G exerts a direct suppressive effect on CD4^+^ T-cells (40) and induces apoptosis in CD8^+^ T-cells (81). A soluble form of HLA-G also participates in the vascular remodeling of maternal uterine spiral arteries during pregnancy (81). Defective HLA-G expression has been associated with PE (82). HLA-G levels in plasma from women who subsequently develop PE are lower than control patients (83, 84). MSC have been shown to secrete and express HLA-G (73, 74).
- *PGE_2_*: it has been postulated that MSC immunosuppression is also mediated by PGE_2_ (85). PGE_2_ is a bioactive lipid synthesized by cyclooxygenase (COX) enzyme pathway. It elicits a wide range of effects on inflammation process and immune cells. PEG_2_ inhibits IFN-g production in CD4^+^ T-cells, which facilitates development of Th2 cytokine production (86), induces the expression of inhibitory receptors on cytotoxic lymphocytes (87), regulates Th17 differentiation and enhances Th17 cytokine expression (88). PGE_2_ also has an effect on innate immune response suppressing proliferation, cytokine secretion, and NK cell-mediated cytotoxicity (89). PGE_2_ is produced by decidua and fetal membranes, and is believed to play a role in the onset of labor (90). Secretion of PGE_2_ by MSC inhibits inflammation (91) and alters T-cell and NK cell proliferation and cytokine production (92) in effector immune cells. However, the evidence of the involvement of PGE_2_ in the development of PE is poor.

All the immunosuppressive properties of MSC have mainly been studied using BM-MSC. However, it has been shown that these cells have different immune behavior than eMSC (93), suggesting that these two MSC types differ in their immunomodulatory and anti-inflammatory effects. Those results converge toward positioning the eMSC as a crucial endometrial cell type that might have a role in uterine physiology and pregnancy. In order to understand the role of maternal immunotolerant mechanisms and how an alteration in these mechanisms could trigger the development of PE, it would be important to isolate and characterize the immune properties of eMSC. For this, further experimental evidence is needed to unravel the functional role of MSC from endometrial origin, the decidua, and in a pregnancy-associated environment, and the possible alterations that could be related to the development of PE.

## Conclusion

The physiology of the immune interaction between the fetus and the mother during pregnancy is an unexplored field that has received increasingly attention during the past years. The understanding of immune interactions during normal pregnancy could help guide the research of pregnancy-associated disorders such as PE that finally allow the development and implementation of effective therapeutic tools. In this regard, the study of MSC biology as master immunomodulatory cell, specifically eMSC, might become an important contribution to the understanding of physiological and pathological immune interactions during the establishment and maintenance of pregnancy that could be related to the development of disease states, such as PE.

## Conflict of Interest Statement

The authors declare that the research was conducted in the absence of any commercial or financial relationships that could be construed as a potential conflict of interest.
